# Arsenic and Its Compounds

**Published:** 1863-01

**Authors:** 


DENTAL REGISTER OF THE WEST.

VOL. XVII.] JANUARY, 1863. [NO. 1.

### Original Essays and Communications.

### ARSENIC AND ITS COMPOUNDS.

(Continued from page 354, of VOL. XVI.)

IN a former paper it was stated that arsenious acid is the best known and most important of the compounds of arsenic. As it is very poisonous, not costly, and in considerable demand for legitimate uses, accidental as well as criminal poisoning with it is common. Hence great attention has been paid by chemists and toxicologists to the means of detecting its presence. Some of these will now be noticed.

TESTING FOR ARSENIOUS ACID. — This subject will be better understood by dividing it, according to Pereira, into a consideration of the characteristics of the solid acid, of an aqueous solution of it, and of its combinations and mixture with organic substances.

*Solid arsenious acid*, when placed on ignited charcoal, is decomposed, its oxygen combines with the carbon, and the metal escapes in the form of vapor. This vapor is barely visible, and possesses a *garlic odor*. This odor is indicative of *metallic arsenic*. The oxygen of the atmosphere soon combines with the metal, liberated by the carbon, and *arsenious acid* is again formed. This appears as a dense, white smoke, without odor.

*The reduction test.*—The fact that ignited carbonaceous matter decomposes arsenious acid is the basis of the reduction test. When the acid is mixed with recently ignited but cold charcoal, and then heated in a test-tube, it is decomposed, and the metallic arsenic is converted into vapor, and condenses on the cooler portions of the tube, forming a bright metallic crust. The outer surface of this crust exhibits the metallic lustre very distinctly, while its inner surface is grayish and crystalline. As arsenicum is quite volatile, this crust may be chased up and down the tube by the flame of the spirit lamp; and by these repeated sublimations, it is so exposed to the atmospheric oxygen as to be again converted into arsenious acid, which adheres to the sides of the tube in the form of octohedral crystals.

This manipulation is conducted more pleasantly when the charcoal is not finely pulverized.

*An aqueous solution of arsenious acid* may be recognized by a variety of tests.

*Ammonio-nitrate of silver* is prepared by adding, drop by drop, aqua ammoniæ to a solution of nitrate of silver. The oxyd of silver is at first precipitated and again taken up or dissolved. The ammonia should be added till the oxyd is *nearly all* redissolved. This test should be prepared with special care; for if too much ammonia is added it will not occasion a precipitate with arsenious acid; and if too little it will cause a precipitate with phosphate of soda, which might deceive the casual observer. Hence, the test should always be tried with both arsenious acid and phosphate of soda before reliance is placed in it.

When the ammonio-nitrate of silver, as thus prepared, is added to a solution of arsenious acid, the *arsenite of silver* is precipitated, and nitrate of ammonia remains in solution. The reactions may be represented thus:—

$$As\,O_3 + AgO,\,NO_5 + NH_3 = Ag\,O,\,AsO_3 + NH_3\,,\,NO_5\,.$$

The arsenite of silver, thus precipitated, is a yellow substance, soluble in aqua ammoniæ, nitrate of ammonia, and nitric acid.

*Ammonio-sulphate of copper* is prepared by adding, as above, aqua ammoniæ to a solution of sulphate of copper till *all* of the oxyd of copper, at first precipitated, is redissolved. Too much ammonia must not be used, and the solution must be considerably diluted. If these precautions are neglected, no precipitate will be obtained. The test should be tried with a known solution of arsenious acid before it is relied on.

When properly prepared, the ammonio-sulphate, added to a solution of arsenious acid, gives a pale green precipitate, which is *arsenite of copper*, (Scheele's green.)

*Hydro-sulphuric acid*, often called *sulphuretted* hydrogen, gives, from a solution of arsenious acid, a yellow precipitate called sulpharsenious acid, ($As\ S_3$.)

The arsenical solution should be slightly acidulated with hydrochloric acid before applying this test; or, if it is already acid, it should be neutralized by an alkali, and then acidulated as above. The hydro-sulphuric acid is conveniently prepared by putting a small quantity of sulphide of iron in a Florence flask or common bottle, and pouring some oil of vitriol on it. The acid begins to escape immediately, and may be passed through the arsenical solution by adapting a perforated cork and bent tube to the flask or bottle, and dipping the free end of the tube into the solution. The precipitate may be dried, and the "reduction test" resorted to for further confirmation.

The sulphide of iron is conveniently prepared by heating together, in a common crucible, 28 parts of iron filings, and 16 of sulphur.

*Nascent hydrogen.*—When hydrogen is liberated in contact with arsenic, or any of its compounds, it combines with the metal, forming a gaseous compound called *arseniuretted* hydrogen, which has been already referred to. This fact was first taken advantage of, in testing for arsenic, by Mr.

Marsh, of England; and, accordingly, the nascent hydrogen is called "Marsh's test."

Various forms of apparatus have been contrived to facilitate the application of this test. The experimenter need never be at a loss; for a common bottle, a perforated cork, and a small tube of glass, metal or pipe-stem will answer the purpose, and, though less convenient than some other appliances, are just as reliable. This process may be conducted as follows:

Place some strips or fragments of pure zinc in the bottle, pour on them some dilute sulphuric acid, add a small quantity of the suspected liquid, and insert the cork with its tube in place. Hydrogen gas is liberated by the action of the zinc on the dilute acid, and, after allowing it to expel all the air from the bottle, it may be ignited at the point of the tube. If no arsenic is present, only the ordinary hydrogen flame will be seen. But if arsenic is present the flame will be bluish-white, and a light colored smoke is evolved. If now a piece of glass, or better still, a piece of white porcelain be held in the flame, so as partly to arrest the combustion, a dark spot, composed of metallic arsenic, will be deposited on it. If held a little higher, arsenious acid will be deposited on it.

*Metallic copper*, when boiled in an acidulated solution of arsenious acid, becomes covered with metallic arsenic, in the form of a steel-gray crust. This is called Reinsch's test, and is very delicate. The copper should be in the form of foil or fine gauze.

These tests are all simple, easily applied; and their combined testimony can not be disputed. Nevertheless, many important precautions must be taken into careful consideration. The *impediments* to their application must be overcome; and their *fallacies* must be guarded against. It will be profitable to notice some of these before considering the detection of arsenic in organic mixtures.

The *garlic odor* test is of but little practical importance.

The combustion of accompanying organic matters often disguises it; and the sense of smell is too variable in its powers to be relied on. This test, too, is liable to the *fallacy* that other substances during combustion emit a similar odor.

*The reduction test* is, in the main, satisfactory. When the character of the crust is doubtful, cut off the part of the tube that contains it, and break it in a mortar fine enough to be introduced readily into another tube, and again apply heat. A very careless observer might mistake a charcoal for an arsenical crust, and one less careless might be deceived by a thin deposit of mercury. The latter metal can be made to coallesce, so as to form a globule, by means of an excavator or the point of a knife. But the arsenical crust can be distinguished from all others by oxydizing it and applying other tests. It is readily oxydized by cutting off the lower end of the test tube, and in heating the crust while the tube is held in an inclined position; and this should never be neglected in medico-legal investigations.

The *impediments to Hume's test*, (ammonio-nitrate of silver,) are chlorides, free acids, and organic matters. If a soluble chloride, as common salt, is contained in the solution, this test throws down a *white* precipitate, the chloride of silver, even when arsenic is present in considerable quantities. The acids may be neutralized by alkalies, and the chlorides may be got rid of by adding nitric acid, and then an excess of the nitrate of silver, and the insoluble chloride of silver may be removed by filtration. The removal of organic matters will be noticed at another place. This test, prepared as above directed, is in no way *fallacious*, as it gives a yellow precipitate with arsenious acid, and with no other known substance. But the white chloride of silver, referred to above, if seen through a yellow liquid, might deceive a careless observer.

*The ammonio-sulphate of copper* test is impeded in its characteristic action by the presence of vegetable astringents, such as tannin, green tea, etc. It is open too to the objection that some organic fluids give a yellow color, and a slight precipitate when no arsenic is present.

*The hydro-sulphuric acid* test alone might deceive, for compounds of antimony, salts of cadmium, and per salts of tin occasion precipitates with it, approximating in color that produced by arsenious acid. The *reduction test* should always accompany this; and the combined testimony of the two is conclusive. In reducing this precipitate, the charcoal should be mixed with dry carbonate of soda.

The presence of organic substances impedes the action of *Marsh's test* by causing the tube or jet to choke up with froth. The way to get rid of this difficulty will be pointed out directly.

This test, too, is liable to several *fallacies*. The sulphuric acid, or the zinc, or even both may contain arsenic. The presence of antimony in the solution will give results similar in appearance to those produced by arsenic. The antimonial crust is darker, and shows less metallic lustre. The arsenical crust is more volatile than the antimonial; and on the basis of this fact Maclagan suggests a method of discriminating between the two. By placing the tube, containing the crust, in olive oil, and heating the oil to its boiling point, an arsenical crust is entirely volatilized, while an antimonial one remains unchanged. In preparing for the olive oil bath, the *Marsh test* apparatus should be slightly different from that described above. The arseniuretted hydrogen should be made to pass through a horizontal glass tube, and a small portion of the tube should be heated by a spirit lamp. The escaping gas is decomposed; and metallic arsenic forms a crust on the cooler part of the tube. One end of the tube should now be sealed up, and it is ready for the oil bath; or a small tube may be used over the flame, instead of the porcelain, as above suggested.

To examine a very faint crust, the tube should be held against a piece of white paper. The crust should also be oxydized, and the liquid tests resorted to.

*Reinsch's test* also requires several precautions. Antimony, silver, platinum, bismuth, and a number of other metals are precipitated on the copper, in this test, as well as arsenic. To be satisfactory, therefore, the reduction test must follow it. According to Christison, the suspected liquid should be mixed with one-tenth of its volume of hydrochloric acid, and heated to its boiling point before the copper is introduced, "otherwise the metal may become tarnished, though no arsenic be present." Inexperienced manipulators seldom allow this test sufficient time. The copper may sometimes be boiled ten, fifteen, or twenty minutes before a visible deposit takes place. And even if the deposit fails to appear, we have not absolute proof that arsenic is not present; for various salts of mercury and other metals render the separation of arsenic by this process difficult, if not impossible.

The combined testimony of these various tests may be positively relied on; and the evidence of all of them should be obtained in every medico-legal investigation where poisoning by arsenic is suspected. No one should begin a research and not make it thorough. The investigator can not afford to be in doubt; and character always, and often life, is in jeopardy.

*Arsenious acid in organic mixtures.*—To prepare an organic mixture containing arsenic for the application of Marsh's test, add to the mixture one fourth of its weight of sulphuric acid, (of course previously tested for arsenic,) and heat in a porcelain vessel, stirring the mixture constantly with a glass rod till the organic matter is charred and nearly dry. Let it cool, and add to it a little nitric acid, which converts the arsenious into arsenic acid. Then evaporate to dryness, and treat the residue with boiling water, and introduce the liquid into the Marsh apparatus. The advantage gained by reducing arsenious acid to arsenic is, that the latter is far more soluble than the former.

For Reinsch's test, boil the organic mixture in one part of hydrochloric acid to eight or ten of water, for two or three hours. Strain the liquid, pressing the residue; and if the quantity be large, concentrate by evaporation. Introduce the copper foil or gauze, and boil for an hour. If a deposit is formed, apply the reduction test, then reoxydize and apply Marsh's test.

